# Potential use of repetitive transcranial magnetic stimulation in treating pediatric avoidant/restrictive food intake disorder: a case report

**DOI:** 10.3389/fpsyg.2025.1593665

**Published:** 2025-07-23

**Authors:** Ru Wang, Shiling Wu, Cuiyuan Fu, Kun Li

**Affiliations:** ^1^Shandong Daizhuang Hospital, Jining, China; ^2^Jining Key Laboratory of Neuromodulation, Jining, China; ^3^Department of Psychology, University of Chinese Academy of Sciences, Beijing, China

**Keywords:** avoidant/restrictive food intake disorder, repetitive transcranial magnetic stimulation, children, efficacy, safety

## Abstract

**Background:**

Avoidant/Restrictive Food Intake Disorder (ARFID) is a heterogeneous eating disorder that typically manifests during adolescence, potentially leading to various health issues, such as malnutrition, developmental delays, and psychological disturbances. Currently, the management of ARFID is multidisciplinary, involving dietary modifications, pharmacological treatments, and psychotherapy, but no standardized treatment protocol exists. Repetitive transcranial magnetic stimulation (rTMS), a non-invasive brain stimulation technique, has shown promise in treating various psychiatric disorders. However, its application in ARFID treatment remains under-explored.

**Case Report:**

This case study presents a 5-year-old boy diagnosed with ARFID and severe malnutrition, who underwent adjunctive low-frequency rTMS therapy. The patient received 1 Hz rTMS stimulation targeting the right dorsolateral prefrontal cortex, with a single session consisting of 1,200 pulses administered once daily for 11 consecutive days. After 11 sessions of TMS treatment, when evaluated using the clinical global impression-improvement scale, the patient was scored 2 points, indicating a moderate improvement in symptoms. Twelve days after hospital admission, the patient exhibited significant improvements in emotional status and eating behavior, and no adverse reactions were observed. Subsequently, the patient was discharged from the hospital. Within two months post-discharge, the patient’s body weight was restored and remained stable throughout the one-year follow-up period.

**Conclusion:**

This case report offers preliminary evidence regarding the application of low-frequency rTMS directed at the right dorsolateral prefrontal cortex as a potential therapeutic approach for childhood ARFID. Our findings add to the burgeoning body of literature on rTMS therapy for ARFID and lend support to the effectiveness and safety of low-frequency rTMS as a treatment modality for childhood ARFID.

## 1 Introduction

Eating disorders (EDs) encompass a range of conditions, including anorexia nervosa, bulimia nervosa, binge eating disorder, avoidant/restrictive food intake disorder (ARFID), other specified feeding or eating disorders, and unspecified feeding or eating disorders (Monteleone et al., 2022). ARFID is a subtype of eating disorders that, unlike other types, does not involve concerns about body image. Instead, it is characterized by one or more of the following three features: diminished appetite, sensory aversions to food, and a heightened fear of negative consequences related to eating, such as choking (Bryant-Waugh, 2019). Sanchez-Cerezo et al. (2023) conducted a review on the prevalence of ARFID and found that studies from specialized eating disorder centers report prevalence rates ranging from 5 to 22.5%. In contrast, research from specialized feeding clinics suggests a higher prevalence, ranging from 32 to 64% (Farag et al., 2022; Krom et al., 2019).

The management of ARFID typically involves a multidisciplinary approach, including dietary interventions, pharmacotherapy, and psychotherapy (Fonseca et al., 2024). However, there is currently no standardized treatment protocol for ARFID, and the focus remains on individualized dietary modifications (Robatto et al., 2024). As the understanding of the neural underpinnings of eating disorders grows, there has been increasing interest in non-invasive brain stimulation techniques, which offer the potential to target brain networks implicated in eating behaviors (Song et al., 2019).

Non-invasive brain stimulation, such as repetitive transcranial magnetic stimulation (rTMS), has gained attention due to its ability to modulate neural activity and improve cognitive and emotional outcomes in various psychiatric conditions (Polanía et al., 2018). The rTMS works by delivering magnetic pulses to the cerebral cortex, which can influence neural plasticity and cortical excitability (Jannati et al., 2023). This technique has demonstrated efficacy and safety in treating anxiety disorders, and conditions such as anorexia nervosa (Guo et al., 2023; Bahadori et al., 2025). Recent evidence suggests that rTMS may also play a role in improving the symptoms of eating disorders by modulating brain networks involved in food intake, appetite regulation, and emotional processing (Aviram-Friedman et al., 2025). The rTMS often targets the dorsolateral prefrontal cortex to regulate disorders such as anorexia nervosa, bulimia nervosa, and binge eating disorder (Longo et al., 2025). Currently, rTMS shows promise in the treatment of EDs, offering additional benefits of minimal side effects and cost-effectiveness (Dalton et al., 2018).

Although non-invasive brain stimulation has been extensively studied in other psychiatric disorders, its application in ARFID, especially in pediatric populations, remains largely unexplored. Studies have shown that from childhood to adulthood, the dorsolateral prefrontal cortex (DLPFC) significantly demonstrates structural and functional maturation in aspects such as cognitive control, emotional regulation, and reward processing (He et al., 2025). Previous studies have primarily focused on high-frequency rTMS protocols targeting the left DLPFC for disorders like anorexia nervosa and bulimia nervosa (Rachid, 2018). However, the right DLPFC (rDLPFC) is characterized by high plasticity and continuously improving functional connectivity, thus showing potential to become an ideal stimulation target for optimizing neural circuits (Wang et al., 2025). Low-frequency rTMS stimulation of the rDLPFC can simultaneously regulate neural mechanisms related to emotion and eating: on the one hand, it can alleviate comorbid anxiety symptoms in ARFID patients and improve anxiety-related restrictive behaviors (Dunlop et al., 2016); on the other hand, the rDLPFC is not only involved in emotional regulation but also closely related to processes such as cognitive control and reward processing (Rolle et al., 2022). For ARFID patients, their abnormal eating behaviors may involve emotional regulation disorders and abnormal perception of food-related rewards (Iron-Segev et al., 2020). Given the high comorbidity of anxiety in ARFID, low-frequency rTMS may offer a novel approach to treating this disorder, potentially enhancing both emotional regulation and eating behaviors. Although the current application of this protocol in the treatment of ARFID is still in the exploratory stage, based on the above theories and relevant research foundations, this protocol has certain rationality and research value.

This case report aims to contribute to the growing body of literature on rTMS therapy for ARFID by exploring the effects of low-frequency rTMS on a pediatric patient diagnosed with ARFID. To our knowledge, this is the first case study to investigate the potential benefits and safety of low-frequency rTMS in treating pediatric ARFID, and it offers preliminary insights into this therapeutic approach.

## 2 Case description

Informed consent was obtained from the patient and his legal guardian prior to the preparation of the following case report.

The patient was a 5-year-old boy who presented with a six-week history of unexplained anorexia, characterized by a significant reduction in food intake, accompanied by periumbilical pain and gastrointestinal discomfort after consuming even small amounts of food. His parents reported that he had progressively developed an increasing reluctance to eat, with an apparent aversion to a wide range of foods. He also exhibited a notable decrease in bowel movements and substantial weight loss, which led to severe nutritional deficits. His parents observed that his emotional state had deteriorated over time, with periods of low energy, increased irritability, and social withdrawal. A stressful family environment was noted, as frequent verbal conflicts between his parents were reported to have contributed to heightened anxiety levels in the child. The worsening of his symptoms was temporally correlated with these domestic conflicts, suggesting a potential psychosocial influence on his restrictive eating behaviors. Before the onset of his symptoms, the patient had been in generally good health, with no prior history of psychiatric illness, chronic disease, or food allergies.

During the six-week period of the disease progression prior to admission, the patient’s weight underwent a significant decline, dropping from 20 kg to 12 kg. Initially, with a weight of 20 kg, his Body Mass Index (BMI) was calculated at 16.5 kg/m^2^, which fell within the normal range corresponding to his age. However, by the time he was admitted to the hospital, his weight had further decreased to 12 kg, resulting in a BMI of 9.9 kg/m^2^. This new BMI placed him well below the expected range for his age group, clearly suggesting a state of severe malnutrition. His energy levels were markedly low, and he displayed signs of general fatigue and decreased physical activity. Although he maintained normal sleep patterns, his overall condition was deteriorating due to insufficient nutrient intake. There was no history of smoking, alcohol use, or substance abuse. Both the patient and his family denied any history of mental illness in the family.

### 2.1 Physical, mental and laboratory examinations

To exclude potential organic causes of his symptoms, the patient underwent a thorough medical evaluation, including a physical examination, laboratory testing, and imaging studies.

#### 2.1.1 Physical examination

During the examination, the patient’s vital signs were stable, and there were no significant differences in the assessment results of the cardiovascular, respiratory, abdominal, and nervous systems.

#### 2.1.2 Psychological and behavioral assessment

During the mental examination, the patient was admitted on a voluntary basis. His facial expression seemed natural, yet he appeared lethargic and were not very cooperative during the examination process. Clinical interviews revealed intact person-place orientation, sustained attention, and relevant responses. Normal orientational functions were noted, though insight into the illness was lacking. Language expression was coherent with appropriate semantic-logical organization. Emotional state was stable, without depression or obsessive-compulsive symptoms. Moreover, his will and behavior did not show any signs of abnormal increase or decrease in willpower, and his sleep–wake rhythm was regular.

According to the Conners’ Parent Rating Scale for Children (Goyette et al., 1978), the T-score for impulsivity-hyperactivity of this child is 43.89, and the T-score for the hyperactivity index is 44.00. Other T-scores include 59.44 for anxiety, 52.61 for psychosomatic disorders, 56.06 for conduct problems, and 47.60 for learning problems. All scores remained within normal ranges.

Using the Hamilton Anxiety Rating Scale (HAMA) (Hamilton, 1959), the patient scored 2 for anxious mood, tension, and fear; 1 for respiratory symptoms; and 3 for gastrointestinal symptoms, totaling 10. A score exceeding 7 indicates possible anxiety.

#### 2.1.3 Laboratory examinations

The results of the patient’s blood routine, liver function, coagulation function, blood lipid, c-reactive protein, and serum electrolytes were all within the normal range. The renal function examination showed a creatinine level of 47.1 μmol/L, which fell within the normal range (44–80 μmol/L), whereas the random blood glucose level of 2.99 mmol/L, below the normal reference range (3.9–6.11 mmol/L), indicating hypoglycemia.

#### 2.1.4 Auxiliary examination

Abdominal ultrasound examination did not reveal any signs of appendicitis, intussusception, or intestinal obstruction. Gastrointestinal ultrasound examination showed no obvious thick-walled or abnormally dilated intestinal loops or abscesses.

Electroencephalogram results were normal, with no signs of epileptiform activity or abnormalities indicative of neurological dysfunction. Electrocardiogram revealed sinus rhythm but showed a prolonged QTc interval, warranting careful monitoring of potential cardiac implications.

### 2.2 Diagnosis

The patient presented with sudden idiopathic anorexia that rapidly progressed to multiple food aversions, accompanied by postprandial periumbilical pain, gastrointestinal discomfort, and subsequent food refusal. Body weight dropped abruptly from 20 kg to 12 kg within 10 days, meeting criteria for extremely severe malnutrition and significant weight loss (WHO, 1999). A psychiatrist’s evaluation confirmed the diagnosis of ARFID (code F50.8) according to the DSM-5 (Call et al., 2013). Through the Structured Clinical Interview for DSM-5, Clinician Version (SCID-5-CV) (First et al., 2016), differential diagnoses excluded major depressive disorder, as the patient lacked core symptoms (anhedonia, feelings of worthlessness, suicidal ideation), and obsessive-compulsive disorder was ruled out due to the absence of obsessive thoughts about food contamination or ritualistic behaviors.

### 2.3 Treatment course

**Figure 1:**
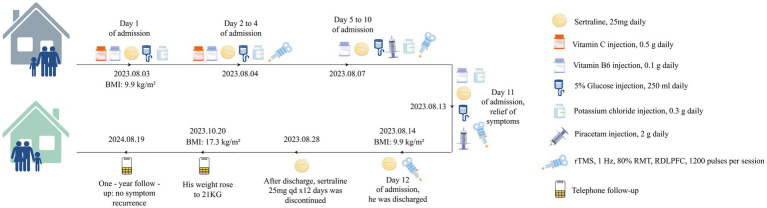
Case summary. rTMS, repetitive transcranial magnetic stimulation; RDLPFC, right dorsolateral prefrontal cortex; ARFID, avoidant/restrictive food intake disorder; RMT, resting motor threshold.

Upon admission, an integrated treatment approach was initiated, combining pharmacological intervention, nutritional support, and rTMS (see Figure 1). Before initiating rTMS treatment, the medical team systematically screened for contraindications and relevant conditions by obtaining an informed consent form signed by the patient’s guardian. Given the patient’s history of progressive food avoidance and emotional instability, a low-dose sertraline regimen (25 mg/day) was introduced to mitigate potential anxiety symptoms that could contribute to his restrictive eating patterns. Simultaneously, intensive nutritional support was provided to stabilize his metabolic status and counteract the effects of prolonged caloric deficiency. Intravenous supplementation included 5% glucose infusion (250 mL/day) to maintain energy levels, vitamin B6 (0.1 g/day) and potassium chloride (0.3 g/day) to correct any micronutrient imbalances, and vitamin C (0.5 g/day) to support overall metabolic function.

On Day 2, low-frequency rTMS was introduced as an adjunct therapy. The TMS was performed using an S-30 model TMS device (Yingchi Company, Shenzhen, China). After localizing the motor cortex area of the contralateral abductor pollicis brevis muscle, the resting motor threshold (RMT) was defined as the TMS intensity at which 5 out of 10 TMS pulses induced motor evoked potentials 50 mV (Zhao et al., 2020). The treatment protocol involved EEG 10–20 system-based localization of rDLPFC (F4), using a pediatric positioning cap, with parameters: 1 Hz frequency, 1,200 pulses/session, 80% RMT, once a day, 11 consecutive days (Pedapati et al., 2015; Smith et al., 2023). After each treatment session, the therapist inquired whether the patient experienced adverse reactions such as dizziness, headache, nausea, ear discomfort, or a burning sensation on the skin, and no discomfort was reported.

The patient demonstrated notable improvements within the first few days of treatment. On Day 5, during ward rounds, inquiry with the patient’s family revealed the patient is no longer prone to irritability, has a reduced frequency of abdominal discomfort during meals, and an increased appetite, which is consistent with the improvement in eating habits and mood stabilization observed. Therefore, the intravenous injection of vitamin C was discontinued, and piracetam (2 g daily) was injected to support brain cell nutrition. Two weeks after the treatment, the patient’s gastrointestinal symptoms and anxiety-related emotions disappeared, and the HAMA score was 0. The patient reported significant relief of physical symptoms, specifically noting the absence of abdominal discomfort during meals and resolution of food aversion. Concomitantly, normal eating habits were restored, as evidenced by the patient’s consistent intake of regular diets without reported distaste or gastrointestinal complaints. However, there was no significant change in body weight, which remained at 12 kg. The Clinical Global Impression-Improvement (CGI-I) scale (Busner and Targum, 2007) score was 2, indicating moderate improvement of symptoms. Subsequently, the patient was discharged from the hospital.

After discharge, the patient received sertraline 25 mg orally daily for 12 days. A telephone follow-up by the attending physician at 2 months showed stable condition, with family reporting regular diet, weight gain to 21 kg (BMI 17.3 kg/m^2^), and no reported discomfort. At 1-year follow-up, parents reported that after discontinuing sertraline (without subsequent treatments), the patient maintained a diverse diet with no symptom recurrence.

## 3 Discussion

The consequences of ARFID are far more serious than those of picky eating, potentially leading to malnutrition, stunted growth, and even psychological disorders that affect the ability to live daily lives (Kennedy et al., 2023). Currently, there is no standardized treatment approach for ARFID, and clinicians often need to refer to therapeutic strategies used for other eating disorders (Fonseca et al., 2024). Current therapeutic strategies are largely multidisciplinary, involving behavioral therapy, dietary modifications, and pharmacotherapy, yet many cases prove resistant to conventional treatments, necessitating the exploration of novel interventions (Wirth, 2023). Recent studies have shown that the brain networks of patients with EDs are frequently abnormal, and non-invasive brain stimulation technology can improve their condition by stimulating these networks (Dunlop et al., 2016). Numerous studies have investigated the use of non-invasive brain stimulation for the treatment of anorexia and bulimia, with most showing promising outcome indicators (Wu et al., 2024).

This case study provides preliminary evidence supporting the potential of low-frequency rTMS targeting the right DLPFC as an adjunctive treatment for pediatric ARFID. While rTMS has been extensively studied in psychiatric disorders such as depression and anxiety, its application in eating disorders remains an emerging area of research (Longo et al., 2025; Cavicchioli et al., 2022). The mechanistic rationale behind rTMS in ARFID lies in its ability to modulate neural circuits implicated in anxiety regulation and food avoidance behaviors (Dalton et al., 2020). Dysfunction in the prefrontal cortex and limbic system has been observed in patients with eating disorders, with evidence suggesting that abnormal activity in the DLPFC contributes to restrictive feeding behaviors and heightened food-related anxiety (Stefano et al., 2013; Chen et al., 2018). By delivering low-frequency stimulation (1 Hz) to the right DLPFC, rTMS may reduce cortical excitability in regions associated with anxiety processing, thereby alleviating food-related fears and restrictive eating behaviors (Longo et al., 2025).

The significant improvement observed in this patient following adjuvant rTMS treatment aligns with previous studies on neuromodulation for eating disorders. While most existing research has focused on high-frequency stimulation of the left DLPFC for conditions such as anorexia nervosa and bulimia nervosa, this study suggests that low-frequency stimulation of the right DLPFC may offer a promising alternative for ARFID, particularly in cases where anxiety plays a central role in food avoidance (Bahadori et al., 2025). Given that ARFID often shares features with anxiety disorders, such as heightened fear of choking, vomiting, or gastrointestinal discomfort, the use of rTMS in targeting neural circuits associated with fear responses and avoidance behaviors is particularly compelling (Eckhardt et al., 2019). In this case, adjuvant rTMS not only facilitated improvements in food intake and eating behavior but also contributed to emotional stabilization, as evidenced by reduced anxiety and increased willingness to eat over time. While rTMS may have played an important role in modulating neural circuits, alleviating anxiety, and improving dietary behavior, concurrent interventions such as pharmacotherapy and intensive nutritional support are also likely to have contributed confoundingeffects to the patient’s recovery.

Another key takeaway from this case is the safety and tolerability of rTMS in a pediatric population. José et al. (2018) examined adverse reactions in children with autism aged 3 to 5 years using intermittent theta-burst stimulation with parameters of 80% MT, 600 pulses, and 20 courses. In this study, the patient tolerated the treatment well, with no adverse reactions reported throughout the 11-day stimulation period. A meta-analysis on safety and efficacy in children and adolescents reported adverse effects such as headache, nausea, and muscle pain, without reporting serious adverse events such as seizures (Qiu et al., 2023). Regarding the incidence of rTMS-induced seizures in children and adolescents, Croarkin et al. (Croarkin and MacMaster, 2019) reported that the incidence is similar to that in adults, not exceeding 0.1–0.6%, and as long as appropriate preventive measures are taken, applying rTMS to children can be considered safe. The absence of significant side effects in this case further supports the safety profile of rTMS for pediatric patients with ARFID, making it a potentially viable treatment option in cases where conventional therapies are insufficient.

In the present case, the patient’s condition failed to exhibit a positive response to the treatments administered in other medical institutions. Given the patient’s juvenile age and the demonstrated potential of low-frequency stimulation targeting the right dorsolateral prefrontal cortex in therapeutic applications, we opted for low-frequency rTMS applied to the right dorsolateral prefrontal cortex as an adjunctive therapeutic approach. Following 11 consecutive days of adjunctive rTMS treatment, a significant amelioration in the patient’s mood was observed. Although the symptoms of ARFID did not manifest immediate improvement during the patient’s hospitalization period, a normal weight gain was recorded within two months post-discharge, and the patient reported no recurrence of the illness during a telephonic follow-up one year later. Consequently, adjuvant low-frequency rTMS stimulation of the right dorsolateral prefrontal cortex is likely to confer benefits in the management of ARFID. Moreover, in prior rTMS treatment regimens for ARFID, the emphasis was predominantly placed on high-frequency stimulation of the dorsolateral prefrontal cortex, whereas low-frequency stimulation was comparatively rare. This case report offers valuable reference for the safe and effective utilization of rTMS in childhood ARFID cases.

### 3.1 Limitations

While this case report highlights the potential efficacy and safety of rTMS for ARFID, several limitations must be acknowledged. First, this study is a single-case report, which inherently limits generalizability. The observed improvements could be influenced by individual patient characteristics, and the lack of a control group prevents definitive conclusions regarding causality. Future studies should explore randomized controlled trials with larger sample sizes to validate these findings and determine optimal treatment parameters for ARFID. Second, no functional neuroimaging (such as fMRI) was conducted to assess pre- and post-treatment changes in brain activity, which could provide insight into the underlying neurobiological mechanisms of rTMS in ARFID. Moreover, diagnosing a 5-year-old patient was challenging due to developmental limitations, which rendered structured interviews and rating scales less reliable. The child’s inability to articulate internalized symptoms, combined with overlapping features of ARFID and obsessive-compulsive disorder, created diagnostic ambiguity that may have affected the specificity of evaluating rTMS therapeutic effects.

Additionally, the long-term sustainability of rTMS-induced improvements in ARFID remains an open question. While the patient in this case maintained stable eating behaviors for one year post-treatment, longer follow-up periods are necessary to assess whether booster sessions of rTMS are required to sustain the therapeutic effects.

## 4 Conclusion

In conclusion, this case report provides preliminary evidence that low-frequency rTMS targeting the right DLPFC may be a safe and effective adjunctive treatment for ARFID in children, particularly for cases characterized by food-related anxiety and restrictive eating behaviors. While further research is needed to confirm these findings, this study contributes to the growing body of literature supporting neuromodulation as a promising therapeutic avenue for pediatric eating disorders. Given the chronic and often treatment-resistant nature of ARFID, exploring innovative and targeted approaches such as rTMS holds significant clinical relevance and may help address critical gaps in current treatment strategies.

## Data Availability

The original contributions presented in the study are included in the article/Supplementary material, further inquiries can be directed to the corresponding author.
